# How Does Stomatal Density and Residual Transpiration Contribute to Osmotic Stress Tolerance?

**DOI:** 10.3390/plants12030494

**Published:** 2023-01-21

**Authors:** Md. Hasanuzzaman, Meixue Zhou, Sergey Shabala

**Affiliations:** 1 Department of Agronomy, Faculty of Agriculture, Sher-e-Bangla Agricultural University, Sher-e-Bangla Nagar, Dhaka 1207, Bangladesh; 2 Tasmanian Institute of Agriculture, University of Tasmania, Hobart, TAS 7001, Australia; 3 International Research Centre for Environmental Membrane Biology, Foshan University, Foshan 528000, China; 4 School of Biological Science, University of Western Australia, Perth, WA 6009, Australia

**Keywords:** abiotic stress, salinity, drought, stomatal density, residual transpiration, crop wild relatives, cuticular wax

## Abstract

Osmotic stress that is induced by salinity and drought affects plant growth and development, resulting in significant losses to global crop production. Consequently, there is a strong need to develop stress-tolerant crops with a higher water use efficiency through breeding programs. Water use efficiency could be improved by decreasing stomatal transpiration without causing a reduction in CO_2_ uptake under osmotic stress conditions. The genetic manipulation of stomatal density could be one of the most promising strategies for breeders to achieve this goal. On the other hand, a substantial amount of water loss occurs across the cuticle without any contribution to carbon gain when the stomata are closed and under osmotic stress. The minimization of cuticular (otherwise known as residual) transpiration also determines the fitness and survival capacity of the plant under the conditions of a water deficit. The deposition of cuticular wax on the leaf epidermis acts as a limiting barrier for residual transpiration. However, the causal relationship between the frequency of stomatal density and plant osmotic stress tolerance and the link between residual transpiration and cuticular wax is not always straightforward, with controversial reports available in the literature. In this review, we focus on these controversies and explore the potential physiological and molecular aspects of controlling stomatal and residual transpiration water loss for improving water use efficiency under osmotic stress conditions via a comparative analysis of the performance of domesticated crops and their wild relatives.

## 1. Introduction

The current trends in climate change are predicted to increase the frequency and severity of drought and salinity, limiting plant growth and productivity globally and ultimately reducing food production, leading to enhanced risks of famine [1,2]. Drought and salinity are arguably the most severe abiotic stresses affecting crop production worldwide [3]. About 10% of cultivated land is affected by salinity and drought globally, reducing the average crop yield by more than 50% [4]. Thus, salinity and drought stress will be the key threats to global food security in the 21st century. At the same time, the world population is predicted to rise to more than 9.9 billion in 2050; this will require an increase in food production by 70% compared to the beginning of the century [5,6]. The early responses of plants to drought and salinity are similar as both stresses result in a physiological drought caused by osmotic stress. In order to survive osmotic stress conditions, plants have evolved a range of integrated biochemical (antioxidant defense system against ROS; de novo synthesis of compatible solutes for osmotic adjustment), morphological (deep root system, leaf rolling, deposition of cuticular wax, and increasing leaf thickness and succulence) and physiological (efficient control of stomatal aperture; maintaining photosynthetic apparatus) mechanisms. Among these physiological and morphological adaptations, the efficient control of stomatal and residual transpiration (RT) to optimize water use efficiency under osmotic stress conditions is arguably the most crucial feature. 

Stomata are the main gateways to both water loss and CO_2_ intake; thus, balancing carbon assimilation and transpiration depends on the fine-tuning of the stomatal aperture. As the ability to gain and assimilate CO_2_ for photosynthesis depends on stomatal conductance, the latter is positively correlated with the yield potential of crops [7]. Thus, stomatal conductance is the key physiological parameter affecting plant productivity under both optimum growth and abiotic stress conditions [7,8,9]. At the same time, water use efficiency is inversely proportional to stomatal conductance. Plants that have a higher stomatal conductance via an increased stomatal density have a higher carbon assimilation rate and faster growth under optimum growth conditions, but they normally show lower water use efficiency and vice versa. On the other hand, increasing water use efficiency is very crucial for plant adaptation under water stress conditions. Stomatal modification can play a significant role in optimizing photosynthesis and water use efficiency under stress conditions. However, stomatal conductance is determined by the physiological adjustment of the stomatal pore area and morphological alterations of stomatal size and stomatal density on the abaxial and adaxial leaf surfaces [10]. Stomatal density and stomatal size are inversely related. A large number of small stomata may also offer greater control of stomatal conductance, as small stomata are considered to be able to adjust stomatal pore area and regulate stomatal conductance more rapidly, thus optimizing water use efficiency over shorter timescales [11,12]. Every plant can rapidly (within minutes) regulate the stomata aperture, but changing density takes days and weeks, so they need to set up “optimal” density and then balance carbon flow/water loss by controlling the aperture.

Stomatal movement is strongly affected by environmental conditions. The effect of osmotic stress on stomatal parameters is complex, with both stomatal conductance and stomatal size and density being affected [13,14,15]. What may bring more adaptive advantages to plants? Fewer but more widely open stomata? Or many stomata with a smaller aperture? The answer to these questions may come from studying stomata movement and patterning in wild crop relatives. These wild relatives have evolved efficient mechanisms to survive under limited water availability [16,17]; however, very little is known about their stomatal patterning under osmotic stress conditions. Do the wild relatives of crops and tolerant genotypes of cultivated crops reduce their stomatal apertures to save water under osmotic stress? Or do they have a superior ability to maintain a constant stomatal movement under stress conditions? In this review, we aimed to investigate the physiological and molecular responses of stomatal movement and patterning under the condition of osmotic stress.

While stomatal conductance is a major regulator of leaf transpiration under normal conditions, water can also be lost from the leaf surface, bypassing stomata through a process known as RT. Through the leaf cuticle, RT could contribute up to 50% of total water loss in stressed plants during the day and 60% during the night [18,19]. It is usually assumed that RT is determined by the total amount of cuticular wax deposited on a leaf surface [20]. It was shown that osmotic stress increased the deposition of cuticular wax on leaf surfaces by up to three-fold [21,22]; therefore, plants can conserve more water by reducing RT while their stomata are closed or partially closed during abiotic stress conditions. This view, however, was challenged by other researchers [23,24,25,26,27]. Thus, the question of whether the total amount of cuticular wax deposition or cuticular wax composition on a leaf surface is responsible for acting as a barrier to control nonstomatal water loss during abiotic stress conditions remains disputed. In this present review, we focused on the role of RT in plant abiotic stress tolerance and the association between RT and cuticular wax biosynthesis during abiotic stresses. 

## 2. Molecular Mechanism of Stomatal Development 

Plants colonized land about 485 million years ago [28]. Stomata have been found in fossils dating back to more than 400 million years ago and are thought to help plants in this process [29]. Stomata are the microscopic epidermal valves that have a critical role in the gas exchange necessary for photosynthetic CO_2_ assimilation along with water loss in plants [30]. Water loss through the stomata also allows plants to regulate leaf temperature. Under drought conditions, stomata protect plants against desiccation through the minimization of water loss. In dicot, the stomata consist of two kidney-shaped guard cells. On the other hand, most grass species’ stomata are formed by two dumbbell-shaped guard cells flanked by two subsidiary cells, although some non-grass monocots have kidney-shaped guard cells and subsidiary cells [28,31]. The development, distribution, morphology, frequency, and positioning of stomata are extremely diversified in different plant species due to their evolutionary adaptation on land. Over the past 20 years, stomatal development has been intensively studied on the model dicot plant *Arabidopsis thaliana*. However, recent studies have been focused on monocot grass, including *Brachypodium distachyon*, *Hordeum vulgare*, and *Oryza sativa,* to investigate the main transcriptional regulators essential for stomatal development [32,33,34]. Stomata are evenly distributed on the leaf epidermis according to the one-cell-spacing rule in most plant species. Stomata develop through a single asymmetric cell division followed by the differentiation of a guard mother cell, which then divides evenly into two guard cells [28]. Two groups of basic helix-loop-helix (bHLH) transcription factors regulate the cellular divisions and cell fate transitions necessary for stomatal development. The first group of bHLH is encoded by the paralogs *SPEECHLESS (SPCH)*, *MUTA,* and *FAMA,* and the second group is encoded by the broadly expressed paralogs *SCRM* and *SCRM2*. These factors are regulated via signaling peptides, including *EPIDERMAL PATTERNING FACTOR* (*EPF1* and *EPF2*), the leucine-rich repeat ERECTA family of membrane receptor kinases (*ER*, *ERL1*, and *ERL2*), and the leucine-rich repeat membrane protein *TOO MANY MOUTHS* (*TMM*) to enforce stomatal spacing and density. Recently, it has been found that *STOMAGEN/EPF-LIKE9* (*EPLF9*) and stomatal *DENSITY AND DISTRIBUTION1* (*SDD1*) act as positive and negative regulators, respectively, which play a role in tissue-specific stomatal patterning. The frequency of stomata on the leaf epidermis is species-specific, but it could be affected by different environmental factors. However, a number of fundamental questions remain unanswered. The most crucial of them is how does stomatal density contribute to osmotic stress tolerance in plants? Answering this question might help to develop stress-tolerant cereal crops for predicted future climate change scenarios. By increasing stomatal density, the plant may be able to increase stomatal conductance and maximize CO_2_ uptake, which could be beneficial for plant nutrient uptake and photosynthesis [35]. It has been shown that osmotic-stress-sensitive barley genotypes naturally contained a higher stomatal density than osmotic-stress-tolerant genotypes under optimum growth conditions [36,37]. Conversely, plants may be able to optimize their water use efficiency by reducing maximum stomatal conductance via a reduced stomatal density [38]. Generally, stress-tolerant barley genotypes have a lower yield performance because of less CO_2_ assimilation and biomass for reduced stomatal density under control conditions, but they have a better survival capacity under hostile environmental conditions than the standard cultivated genotypes because of their increased water use efficiency [14,39]. However, to the best of our knowledge, stomatal density has not been used in any breeding program as a physiological trait to improve osmotic stress tolerance in crops. Before using stomatal density as a targeted physiological index in breeding programs, it is essential to understand whether changes in stomatal density happen as a consequence of reduced leaf area or if it is driven by evolutionary mechanisms for stomatal development for better adaptation under stress conditions. 

## 3. Physiological Response of Stomatal Density to Plant Osmotic Stress Tolerance

Drought and salinity stress alter stomatal density in different plant species, although the reported data are sometimes controversial (Table 1). An increase in stomatal density was observed in *Pseudoroegneria spicata* in response to drought stress [40]. Moderate drought stress increased stomatal density in the leaves of *Leymus chinensis* and *Zea mays* [41,42]. However, leaves that developed under severe drought stress conditions had reduced stomatal density when compared to well-irrigated conditions and showed better drought tolerance in *Leymus chinensis*, *Populus balsamifera*, *Oryza sativa,* and *Triticum aestivum* [41,43,44,45]. It is generally hypothesized that leaves from higher salinity stresses would have a lower stomatal density in order to reduce water loss. This is based on the observations made on halophyte, in which leaf size and stomatal density are reduced under high salinity stress (>400 mM NaCl) conditions. This hypothesis has been confirmed by the follow-up work, which showed that salinity causes a significant (14–34%) reduction in stomatal density in *Chenopodium quinoa* and *Chenopodium album* and represents a fundamental mechanism by which plants can optimize water use efficiency under higher saline conditions [15,46,47,48]. Similar results were found in basil and strawberry under saline environments [49,50]. A reduction in stomatal density with increasing salinity levels was observed in different highly salt-tolerant halophyte species [47]. The reduced stomatal density may delay the accumulation of toxic ions and growth-inhibiting signaling molecules into the leaves through a reduced transpirational stream under salt stress, facilitating salinity stress adaptation [49,50]. The above-observed decrease in stomatal density in halophyte may be explained by the fact that halophytes exhibit higher succulency when grown under high salinity stress and, as a result, the pavement cells of the epidermis increase their cell volume and push stomata further apart, thus decreasing stomatal density. However, stomatal density increased by 23% in response to higher salinity stress in barley [19], with increasing stomatal density showing a positive correlation with salinity tolerance [36]. Follow-up work on 80 barley genotypes was conducted under severe saline conditions for four weeks (300 mM NaCl), and a similar result was found [14]. Traditionally bred rice varieties with a high stomatal density and small stomatal size typically have lower biomasses, and these varieties are more resilient to drought than those with low stomatal density and large stomatal size [51]. Additionally, it was found that stomatal density increased with increasing salinity levels in the *Beta maritima* (sea beet) and *Beta vulgaris* (sugar beet) [15]. 

Phenotypic plasticity is one of the main characteristics of a plant that is used to acclimatize to different growth conditions. The increasing stomatal density found in salinity and drought-induced plants might be due to changes in leaf anatomy. A reduction in leaf size is the most common phenomenon for plants to adjust to salinity or drought stress conditions [41]. Higher salinity and drought stress restricted plant growth and decreased leaf expansion [52]. On the other hand, leaf area reduction is accompanied by an increase in leaf vein and stomatal density [53,54,55,56]. Thus, salinity and drought may increase stomatal density by causing a reduction in leaf size to overcome simple geometric practicalities of fitting enough functional stomata per unit of leaf surface area to meet the desired CO_2_ flux and to service the photosynthetic capacity [57]. However, there was a strong relationship between stomatal size and density [58]. Smaller stomata have faster dynamic characteristics, which has implications for improved long-term water use efficiency and a lower risk of disruption to the leaf hydraulic system [59], allowing the stomata to open and close faster and, thus, respond rapidly to environmental change [58]. However, the correlations between stomatal density, gas exchange, and photosynthesis were not consistent in previous reports. Negative correlations between stomatal density and stomatal conductance, transpiration, and photosynthesis were observed in Mediterranean plants (wheat and maize) [42,60,61]. Conversely, stomatal density was positively correlated with net photosynthetic rate, transpiration rate, and water use efficiency in *Leymus chinensis* [41]. Arabidopsis mutants showed that increased stomatal density enhanced their photosynthesis rate by 30% by modulating gas diffusion [35]. On the other hand, a reduction in stomatal conductance through reduced stomatal density increased water use efficiency without altering photosynthetic capacity [38]. In barley, a reduction in stomatal density increased water use efficiency without affecting plant yield [62]. When taken together, it is clear that increasing stomatal density under salinity or drought stress is a consequence of reduced cell size and leaf area. However, the reduction in stomatal density in newly developed leaves under water stress conditions is a stress tolerance mechanism by which plants can reduce unwanted water loss and optimize water use efficiency for better survival under osmotic stress conditions. Thus, stomatal density could be used as an osmotic stress tolerance trait in breeding programs. 

**Table 1:** Osmotic stress-induced changes in stomatal density in different plant species.

Plant Species	Stress	Stress Level	Response of Stomatal Density	Reference
*Lycopersicon esculentum*	Salinity	70 mM for 8 weeks	Decreased	[63]
*Chenopodium quinoa*	Salinity	400 mM for 8 weeks	Decreased	[47]
*Chenopodium quinoa*	Salinity	400 mM for 7 weeks	Decreased	[46]
*Fragaria ananassa*	Salinity	40 mM	Decreased	[50]
*Hordeum vulgare*	Salinity	200 mM for 4 weeks	Increased	[36]
*Hordeum vulgare*	Salinity	300 mM for 4 weeks	Increased	[14]
*Sporobolus ioclados* *Cymbopogon jwarancusa* *Ochthochloa compressa*	Salinity	300 mM for 8 weeks	Increased	[64]
*Ocimum basilicum*	Salinity	200 mM for 4 weeks	Decreased	[49]
*Laguncularia racemosa*	Salinity	30 mM	Decreased	[65]
*Leymus chinensis*	Drought	Moderate and severe drought	Increased in moderate stress and decreased in severe stress	[41]
*Oryza sativa*	Drought	Severe drought	Decreased	[43,66,67]
*Triticum aestivum*	Drought	Moderate drought	Decreased	[68,69]
*Hordeum vulgare*	Drought	Moderate drought	Decreased	[70]

## 4. Molecular Regulation of Stomatal Density for Osmotic Stress Tolerance

The *EPF*, *SDD*, and *STOMAGEN* or the *EPFL9*, *TMM*, *SPEECHLESS*, *MUTA*, *FAMA,* and *ERECTA* genes have been proven and practically used to regulate stomatal development, distribution, pattern, size, and density on plant leaves (Table 2). The manipulation of the expression level of the *EPF* family, which contains 11 (*EPF1*, *EPF2*, and *EPFL1–9*) genes, has been confirmed to be a powerful tool for modifying stomatal density and patterning. The constitutive overexpression of either *EPF1* or *EPF2* genes results in decreasing stomatal density [71]. A lack of *EPF1* and *EPF2* genes, which are normally expressed in the guard cells of young stomata and their precursors, resulted in an Increase in stomatal density and clustering on the leaf epidermis [71,72]. The double *epf1epf2* mutant of Arabidopsis exhibited a higher stomatal density [38,73]. Water use efficiency could be improved directly by the manipulation of the *EPF* genes controlling stomatal density. A reduction in stomatal density via the overexpression of *EPF1* and *EPF2* genes increased WUE by 20% in Arabidopsis without affecting photosynthesis, and plants lacking both *EPF1* and *EPF2* expression in *epf1epf2* mutants exhibited higher stomatal density, resulting in higher maximum stomatal conductance, with lower WUE [38]. The overexpression of the *HvEPF1* gene in barley significantly reduced their stomatal density and enhanced WUE and drought tolerance without affecting grain yield [62]. The overexpression of *PdEPF1* in transgenic white poplar reduced stomatal density by 28% and increased WUE and drought tolerance by reducing transpiration by 30% [74]. Transgenic Arabidopsis, which overexpressed *PdEPF2,* enhanced its drought tolerance by limiting transpirational water loss as a result of decreasing stomatal density [75]. It was found that the overexpression of *PdEPFL6* regulated the expression levels of MAPK-associated genes and rapidly decreased the expression of the transcription factors related to stomatal development (such as *PdSPCH* and *PdMUTE*) under drought conditions, consequently leading to a reduction in stomatal density. This resulted in a marked improvement in drought tolerance in poplar by limiting transpirational water loss [76]. Plants with a reduced expression of *SDD1* and *TMM1* have increased their stomatal density with a low level of stomatal pairing. *Arabidopsis thaliana* stomatal mutant’s *sdd1-1* and *tmm1* increased their stomatal density when compared to their wild types; the increase in stomatal density was correlated with stomatal conductance [77]. On the other hand, a higher CO_2_ assimilation rate was found in *sdd1* mutants when compared to wild *Arabidopsis* as a result of higher stomatal conductance due to an increase in stomatal density [78]. The overexpression of *ZmSDD1* enhanced drought resistance in transgenic maize plants by reducing stomatal density by 30% compared to the wild-type, thus improving the WUE and photosynthetic rate [79]. The overexpression of *AtSDD1* and *SchSDD1-like* in Arabidopsis and tomato plants decreased the stomatal index and density of the leaves, respectively, and resulted in higher dehydration avoidance [80]. The overexpression of STOMAGEN leads to a two–three-fold increase in stomatal density, resulting in a 30% increase in photosynthetic CO_2_ assimilation due to more CO_2_ diffusion into the leaf [35]. In transgenic Arabidopsis, the overexpression of *MtCAS31* significantly reduced stomatal density and enhanced drought tolerance [81]. The overexpression of the rice epidermal patterning factor *OsEPF1* reduced stomatal density and showed higher drought and temperature tolerance with an improved yield by maintaining stomatal conductance and water use efficiency [67]. Late embryogenesis abundant (LEA) proteins are a large protein family that mainly functions in protecting cells from abiotic stress; however, these proteins are also involved in drought tolerance and the determination of stomatal patterning and density. The overexpression of *LEA13* and *LEA30*, *CaLEA6* and *CaLEA73* (from *Capsicum annuum*), and *MsLEA4-4* (from *Medicago sativa*) genes in *Arabidopsis thaliana* plants increased drought tolerance due to lower transpiration and stomatal density compared to control plants [82]. Homeodomain leucine zipper (HD-Zip) transcription factors are plant-specific TFs that participate in abiotic stress responses, and *MdHB7-like* (an HD-Zip I TF) was identified as a drought-induced gene in apple plants. Transgenic apple with the upregulated expression of *MdHB7-like* reduced stomatal density by affecting the expression of stomatal-development-related genes, such as MdEPFL9.1 and MdEPFL9.2., and reduced transpirational water loss, leading to enhanced drought tolerance [83]. *Medicago sativa* NUCLEAR TRANSPORT FACTOR 2-LIKE (*MsNTF2L*) was identified as a nucleus-, cytoplasm-, and plasma membrane-localized protein. The transcriptional expression of *MsNTF2L* is highly induced by ABA and drought stress. The overexpression of *MsNTF2L* increased drought tolerance in alfalfa via the modulation of leaf water loss by regulating both stomatal density and wax deposition [84].

Extensive genetic and molecular studies using knockout/knockdown mutants and transgenic overexpression lines in different plants have found that many transcription factors belonging to the NAC, AP2/ERF, MYB, WRKY, bZIP, homeodomain, bHLH, NF-Y, and CAMTA families, play important roles in reducing transpirational water loss through stomatal aperture and density under salinity stress conditions. The down regulation of Cys2/His2 zinc finger transcriptional factor *DST* (drought and salt tolerance) function reduced stomatal density resulting in reduced water loss and enhanced salinity tolerance in rice [85]. The overexpression of the *ABP9* gene, encoding a bZIP transcription factor in cotton has improved water conservation by reducing transpiration through reducing the stomatal aperture and stomatal density, thus enhancing salinity tolerance [86]. The overexpression of a stress-responsive transcription factor gene *ONAC022* in transgenic rice genotypes displayed a higher salt tolerance and accumulated less Na^+^ in the root and shoots by reducing transpirational water loss by reducing the percentage of open stomata [87]. The R1-MYB transcription factor encoded by the *ARS1* (altered response to salt stress 1) gene contributes to reducing transpirational water loss by controlling the stomatal aperture under salinity stress conditions [88]. The overexpression of the transcription factor *ZmNAC49* affects the expression of genes related to stomatal development, namely *ZmTMM*, *ZmSDD1*, *ZmMUTE*, and *ZmFAMA,* and significantly decreases the transpiration rate, stomatal conductance, and stomatal density, thereby enhancing drought tolerance in maize [89]. *PdERECTA* overexpression in poplar plants survived longer and performed better within limited water conditions by altering the development pattern of stomata to reduce stomatal density, thereby restricting water consumption and conferring enhanced drought tolerance [90].

**Table 2:** Genes involved in the regulation of stomatal density in plants under osmotic stresses.

Plant Species	Gene Expression	Response to Stomatal Density	Stress Response	Reference
*Arabidopsis thaliana*	*HD-START*	Decreased	Increased drought tolerance	[91]
*Lycopersicon esculentum*	*SchSDD1-like*	Decreased	Higher dehydration avoidance	[80]
*Arabidopsis thaliana*	*AtSDD1*	Decreased	Optimize transpiration and WUE, and increased drought tolerance	[92]
*Populus tremula*	*PtaGTL1*	Decreased	Increased drought tolerance	[93]
*Zea mays*	*ZmSDD1*	Decreased	Increase drought resistance, WUE, and photosynthesis	[79]
*Arabidopsis thaliana*	*AtGTL1*	Decreased	Increased drought tolerance	[94]
*Hordeum vulgare*	*HvEPF1*	Decreased	Increased drought tolerance	[62]
*Oryza sativa*	*ZmSHR1/* *OsSHR2*	Increased	Increased drought tolerance without impact on photosynthesis	[95]
*Arabidopsis thaliana*	*MtCAS31*	Decreased	Enhance drought tolerance	[81]
*Arabidopsis thaliana*	*EPF2*	Decreased	Increased drought tolerance	[96]
*Oryza sativa*	*PHYB*	Increased	Reduced drought tolerance	[43]
*Arabidopsis thaliana*	*PHYB*	Increased	Reduced drought tolerance	[97]
*Arabidopsis thaliana*	*atdtm1 mutant*	Decreased	Increased drought tolerance	[98]
*Arabidopsis thaliana*	*EDT1/HDG11-ERECTA-E2Fa*	Decreased	Improved WUE and drought tolerance	[99]
*Zea mays*	*TPS1*	Decreased	Improved drought tolerance	[100]
*Arabidopsis thaliana*	*EPF2*	Decreased	Improved WUE	[38]
*Arabidopsis thaliana*	*epf1epf2 mutant*	Increased	Reduced WUE	[38]
*Populus tremula*	*PdEPF2*	Decreased	Increased drought resistance and WUE	[75]
*Populus tremula*	*PdEPF1*	Decreased	Increased drought tolerance	[74]
*Populus tremula*	*PdERECTA*	Decreased	Enhanced WUE and drought tolerance	[90]
*Arabidopsis thaliana*	*EPF2*	Decreased	Increased drought tolerance	[96]
*Arabidopsis thaliana*	*STOMAGEN*	Increased	Increased photosynthesis	[35]
*Arabidopsis thaliana*	*ANGUSTIFOLIA3, YODA mutant*	Decreased	Increased drought tolerance and WUE	[101]
*Arabidopsis thaliana*	*sdd1-1 mutant*	Increased	Responsible for stomatal density	[102]
*Zea mays*	*ZmNAC49*	Decreased	Enhances drought tolerance	[89]
*Arabidopsis thaliana*	*CRK33*	Decreased	Decreased transpiration and increased drought tolerance	[103]
*Oryza sativa*	*OsJAZ9*	Decreased	Lower leaf transpiration and enhance drought tolerance	[104]
*Arabidopsis thaliana*	*sdd1 mutant*	Increased	Responsible for stomatal density	[77]

When taken together, this suggests that plants with a lower stomatal density are more stress tolerant and more efficient in their water use under water deficit conditions and would perform better under future climate scenarios.

## 5. Stomatal Movement in Wild Relatives of Crops

The wild relatives of different domesticated crops, such as rice, wheat, barley, and maize (Table 3), are more tolerant to harsh environmental conditions, as, collectively, they possess a unique collection of morphological, anatomical [105,106], and physiological traits [107,108,109] that are still unused for the improvement of cultivated crops. Among the different traits, the efficient control of transpiration through stomata is one of the crucial features of wild relatives for balancing the efficiency of CO_2_ assimilation and transpiration under abiotic stress conditions. Xerophytic barley has low stomatal conductance and is able to survive in arid conditions [110]. These findings suggest that manipulating stomatal density can be a promising way for plant breeders to optimize cereal yields in abiotic stress environmental conditions.

Wild barley (*Hordeum spontaneum*) is an ancestor of cultivated barley, which has evolved efficient mechanisms to survive in severe environmental conditions [111,112] and has a wide range of adaptability to drought, salinity, and extreme temperatures and stress conditions [113,114]. Wild wheat and barley genotypes generally have a better survival capacity when exposed to extreme saline and drought conditions when compared to cultivated wheat and barley. The productivity of wild barley is hardly satisfactory, even under control conditions. Hence, stomata density might be one of the constraints causing yield penalties in wild barley genotypes. Under optimum growth conditions, wild barley shows lower stomatal density when compared to cultivated barley [36]. However, a 16% reduction in stomatal density was found in cultivated barley varieties under moderate to severe saline conditions (200–300 mM NaCl), while the stomatal density of wild barley plants remained unchanged [36,39]. Wild barley plants maintain constant stomatal density under saline conditions to maximize photosynthesis. Wild barley plants have faster stomatal regulation and superior stomatal conductance under salinity stress conditions. 

The rice family consists of 24 *Oryza* species, of which two are domesticated (*O. sativa* and *O. glaberrima*), and the others are commonly known as wild rice or nondomesticated rice. Studies conducted by Chatterjee et al. [115] and Kondamudi et al. [116] characterized the stomatal density of a total of 23 *Oryza* species under optimum growth conditions and revealed that stomata density varies significantly among different *Oryza* complexes. Based on their findings, it was observed that the average stomata density is lower in wild rice when compared to Asian and African cultivated rice. Moreover, it was noticed from their study that stomata vary from very small (higher stomatal density) in *O. nivara*, *O. meridionalis*, *O. granulate,* and *O. meyeriana* to (relatively) very big (lower stomatal density) in *O. grandiglumis*, *O. ridleyi*, *O longiglumis*, and *O. coarctata*. 

A study conducted by Shahzad et al. [117] with six rice cultivars (*Oryza sativa* L.) and four wild rice species accessions (*O. alta*, *O. barthii*, *O. australiensis*, and *O. punctata*) under moderate (50 mM NaCl) to severe (100 mM NaCl) salinity stress showed that wild rice species possess a better ability to control gas exchange as a means of coping with salinity stress. This could be due to the fact that wild rice naturally possesses lower stomatal density, with larger stomata size. 

## 6. Residual Transpiration Is a Component of the Osmotic Stress Tolerance Mechanism

Stomata and cuticles on the leaf epidermis allow plants to maintain a favorable water status. The efficient control of both stomatal and cuticular water loss is a prerequisite for plants to survive under hostile environmental conditions. When plants open their stomata to uptake CO_2_ for photosynthesis, they typically lose 97% of the absorbed water to the atmosphere through the stomata under optimum growth conditions. When the water deficit is increased enough to induce stomatal closure (to minimize the water loss and avoid osmotic stress), the only way for water loss to occur is through the leaf surface to the atmosphere across the cuticle of the leaves. Under such conditions, cuticular transpirational water loss may account for a considerable amount of the daily water used by plants. Cuticular water loss typically amounts to 5–15% of stomatal transpiration, yet it can exceed 50% depending on the environmental conditions and plant species [118]. It is generally assumed that stomata close tightly under dark conditions or at night, but some water can escape from the leaf via stomata even when they are fully closed [119]. Because of this, the water loss through the cuticle at a minimal stomatal aperture under dark conditions is not always (technically) correctly termed cuticular transpiration. Under this condition, RT at minimum stomatal conductance could be a proxy for cuticular transpiration. Under osmotic stress conditions with the stomata closed, the fitness and survival capacity of plants depends on the restriction of RT. Thus, plants that have a lower RT capacity can conserve a higher relative water content under water stress conditions. Therefore, RT could be a potentially useful mechanism for improving plant performance by conserving water, and it could be a selection criterion in cereal breeding programs focused on dry environments. Recently, it has been suggested that RT is a fundamental mechanism of salt tolerance through which plants can optimize water use efficiency under salinity stress conditions [120]. RT water loss per unit leaf area of barley was reduced by 71% under 150 mM NaCl [19]. It was found that RT was reduced by 25–80% in barley when the plants were exposed to 300 mM NaCl for four weeks, and the plant salinity damage index was positively correlated with RT, indicating that the salinity-tolerant genotypes were more efficient in reducing RT than the salinity-sensitive genotypes [14]. It was reported that RT was 20% lower in barley under drought-stress conditions than under well-irrigated conditions [121]. RT also plays an important role in crop yield and is negatively correlated with crop yield. It has been observed that barley, wheat, and alfalfa genotypes that have a lower RT adapted and performed better yields under drought stress conditions [118,121,122,123]. Thus, it appears that under osmotic stress conditions, reducing RT may be an important determinant of WUE and a potential mechanism for improving plant performance.

## 7. Residual Transpiration and Cuticular Wax Deposition

**Figure 1:**
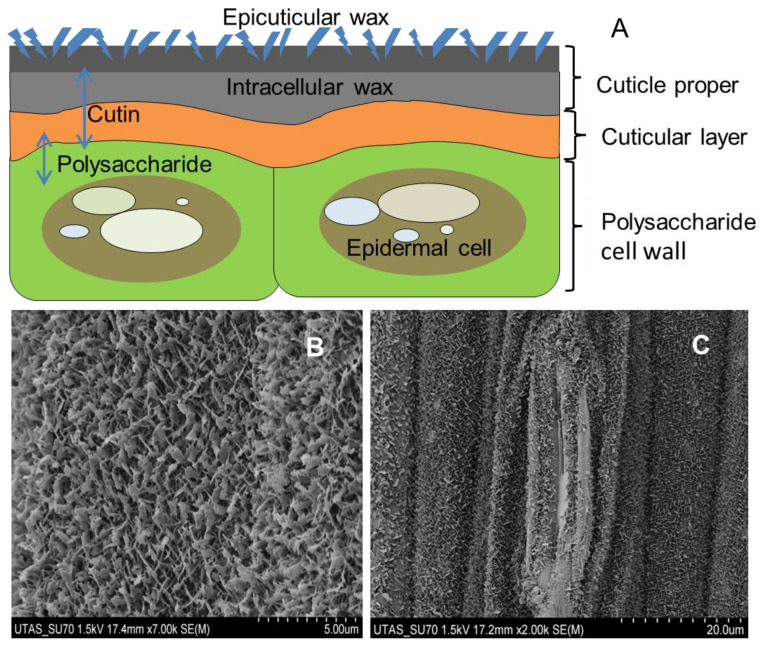
Plant cuticle structure: (**A**) A schematic diagram of plant cuticle, highlighting the major structural features of the cuticle and the underlying epidermal cell layer: cuticle proper, consisting of epicuticular wax, epicuticular wax films, intracuticular wax, and cutin; cuticular layer, consisting of intracuticular waxes, cutin, and polysaccharides; (**B**) scanning electron micrograph image of epicuticular wax film of barley; (**C**) Scanning electron microscopic image of epicuticular wax with stomata of barley leaf surface.

The total amount of epicuticular wax in the cuticle of the leaf increased under different environmental stress conditions, such as high temperature, drought, and UV radiation; thus, plants can increase their water use efficiency and adapt to stress conditions by reducing RT (Table 4). The cuticle is a thin continuous hydrophobic extracellular polymer membrane of the aerial plant parts, consisting of an insoluble polymer matrix known as cutin and soluble cuticular lipid known as cuticular waxes and polysaccharides. Cutin is a three-dimensional polyester-type biopolymer composed of two families of hydroxy and hydroxyepoxy fatty acids. According to histochemical staining and chemical analysis, the microscopic structure of the cuticle is often divided into two domains: one is the cuticular layer, and the other is the cuticle proper (Figure 1). The cuticle layer is a cutin-rich domain with embedded polysaccharides and an overlying layer that is less abundant in polysaccharides but is enriched in waxes, referred to as the cuticle proper (Figure 1). The waxes are either deposited within the cutin matrix, known as intracuticular wax or accumulate on its surface, known as epicuticular wax crystals or films. Epicuticular waxes are defined by their ability to be mechanically removed from the surface of a tissue, whereas intracuticular waxes constitute the leftovers after the mechanical removal of epicuticular waxes [124,125]. Cuticular waxes consist of a complex mixture of very-long-chain fatty acids, primary *n*-alcohols, secondary *n*-alcohols, *n*-aldehydes, *n*-alkanes, *n*-alkyl esters, and cyclic organic compounds, like pentacyclic triterpenoids, flavonoids, tocopherols, and hydroxycinnamic acid derivatives. It was found that epicuticular wax was chemically different from intracuticular wax. The intracuticular wax is more dominated by triterpenoids, whereas long-chain aliphatic molecules are present more equally in bothepi and intracuticular waxes [126]. The functions of cuticular waxes include limiting transpirational water loss, defending against an attack by insects and pathogens, reflecting UV-radiation, reducing water retention on the plant’s surfaces by controlling surface wettability, providing a selfcleaning mechanism, controlling the loss and uptake of polar solutes, and regulating the exchange of gases and vapor [127]. Among these, cuticular wax acts as a barrier to RT and plays an important role in protecting plants against abiotic environmental stresses [125]. It is a common statement in textbooks that the waxes constitute the main water-transport-limiting barrier of the cuticles, especially when stomata are closed. The hypothesis is based on the extraction of cuticular waxes from the plant epidermis with an organic solvent that leads to an increase in cuticular water permeability by one–two-fold [128]. Some literature has suggested that plants that have thicker cuticles or a cuticle containing a higher amount of wax are more efficient in reducing RT [120,121,129,130]. This view, however, was challenged by other researchers who have shown that an increase in the amounts of cuticular wax in the leaf did not lead to decreased rates of RT [20,25,26,27,130]. Therefore, increased amounts of cuticular wax due to environmental stress raises the question of whether the epicuticular or intracuticular wax fraction contributes to the formation of the RT barrier. It has been reported that epicuticular wax does not contribute to the formation of the transport barrier of leaves, whereas the main portion of the transpiration barrier is located in the intracuticular wax layer [124,131,132]. The cuticular transpiration barrier depends on the chemical compositions of the two cuticular fractions [26]. The chemical composition of the epicuticular and intracuticular waxes differ significantly among different species [26,124]. It was found that the RT barrier is associated mainly with very-long-chain fatty acid derivatives, such as alcohols, alkyl esters, aldehydes, and alkanes, and less with alicyclic wax constituents [26,120]. When taken together, it was concluded that the RT barrier is essentially established by the intracuticular wax fraction if the cuticular wax exclusively consists of long-chain aliphatic wax molecules. If both cyclic triterpenoids and long-chain aliphatic molecules consist of cuticular wax, then both the epi- and intracuticular wax fractions contribute to the formation of the transpiration barrier [26].

## 8. Conclusions

Osmotic stress observed in nature under saline and drought stress conditions affects plant growth and productivity in major crops. Therefore, future food security cannot be achieved without a major breakthrough in crop breeding for osmotic stress tolerance. Both stomatal density and RT are crucial for optimizing water use efficiency under osmotic stress conditions. A reduction in stomatal density and RT are critical traits that assist with plants’ adaptation to saline and drought conditions by allowing them to conserve more water. Therefore, plant breeders could use stomatal density and RT as functional markers to select contrasting varieties and create DH lines following the QTL mapping of these traits. Many genes have been identified as the basis of the molecular framework for controlling stomatal development, pattern, and distribution. Consequently, the alteration of specific genes that determine stomatal patterning and distribution could be incorporated to progress breeding for osmotic stress tolerance with the help of genomic sequences and gene editing tools like the CRISPR-CAS9 system. Breeders can also identify the genes responsible for stomatal frequency from the stress tolerance wild relatives of crops. Cuticular waxes also act as a barrier to RT. Until now, several cuticular wax-related genes have been found that are responsible for optimizing water use efficiency for drought tolerance. Further progress in this field may allow for the use of modern genetic tools to modify cuticle-related traits to improve stress tolerance in crops.

## Figures and Tables

**Table 3:** Wild relatives of different crops.

Crops	Crop Wild Relatives
Rice (*Oryza sativa*)	*Oryza glaberrima* *O. barthii* *O. rufipogon* *O. longistaminata* *O. nivara* *O. glumaepatula* *O. meridionalis* *O. officinalis* *O. rhizomatis* *O. punctata* *O. australiensis* *O. grandiglumis* *O. eichingeri* *O. alta* *O. minuta* *O. latifolia* *O. granulate* *O. meyeriana* *O. ridleyi* *O. longiglumis* *O. coarctata* *O. brachyantha* *O. schlechteri*
Wheat (*Triticum aestivum*)	*Triticum monococcum* *T. dicoccoides* *T. urartu* *Aegilops tauschii* *T. turgidum*
Barley (*Hordeum vulgare*)	*Hordeum spontaneum* *H. arizonicum*
Maize (*Zea mays*)	*Zea diploperennis* *Z. huehuetenangensis* *Z. nicaraguensis* *Z. luxurians* *Tripsacum dactyloides*
Sorghum (*Sorghum bicolor*)	*Sorghum halepense*
Oats (*Avena sativa*)	*Avena byzantina*
Quinoa (*Chenopodium quinoa*)	*Chenopodium berlandieri* *C. album* *C. carnosolum* *C. petiolare* *C. pallidicaule* *C. hircinum* *C. ambrosioides* *C. incisum*

**Table 4:** Genes involved in cuticular wax biosynthesis in plants under osmotic stress conditions.

Crop	Gene	Response on Cuticular Wax	Stress Response	Reference
*Medicago sativa*	*WXP1*	30–38% increased the total cuticular wax, especially C30 primary alcohol	Reduced water loss and chlorophyll leaching, delayed wilting, quick recovery after rewatering, and increased drought tolerance	[133]
*Arabidopsis thaliana*	*SHN*	6-fold increase in total cuticular wax and reduced chlorophyll leaching	Increased drought tolerance and recovery	[134]
*Arabidopsis thaliana*		Increased vary-long-chain fatty acid and total cuticular wax	Increased drought resistance	[135]
*Nicotiana glauca*	*LTP*	Total cuticular wax load increased by 1.5- to 2.5-fold	Increased drought tolerance	[136]
*Arabidopsis thaliana*	*WXP1* and *WXP2*	Increased cuticular wax deposition	Improved plant drought and freezing tolerance	[137]
*Arabidopsis thaliana*	*EsWAX1*	Increased the accumulation of cuticular wax	Improved plant drought tolerance	[138]
*Arabidopsis thaliana*	*WIN1/SHN1*	Increased the cuticular wax accumulation and reduced stomatal density	Increased drought tolerance and WUE	[139]
*Oryza sativa*	*Glossy 1(GL1)*	Increased total cuticular wax	Increased drought tolerance	[140]
*Camelina sativa*	*MYB96*	Deposition of epicuticular wax crystals and total wax loads increased, increased levels of alkanes and primary alcohols	Increased drought resistance	[141]
*Oryza sativa*	*OsGL1-6*	Decreased leaf cuticular wax deposition, thinner cuticle membrane, increased chlorophyll leaching and water loss rates	Enhanced drought sensitivity	[142]
*Lycopersicon esculentum*	*SlSHN1*	Higher cuticular wax deposition on leaf epidermal, improved WUE, and reduced water loss rate	Enhance drought tolerance	[143]
*Arabidopsis thaliana*	*GsWRKY2*	Increased epicuticular wax crystals and a much thicker cuticle, less chlorophyll leaching	Enhancing drought tolerance and regulating ABA signaling	[144]
*Agrostis stolonifera*	*Osa-miR319a*	Increased leaf wax content and water retention	Enhanced drought and salt tolerance	[145]
*Oryza sativa*	*DWA1*	Increased cuticular wax, especially very-long-chain fatty acids	Increased drought resistance	[146]
*Oryza sativa*	*OsWR1*	Increased wax synthesis through the alteration of long-chain fatty acids and alkanes	Reduced water loss and enhanced drought tolerance	[147]
*Oryza sativa*	*osgl1-1 mutant*	Decreased cuticular wax deposition, thinner cuticular membrane, decreased chlorophyll leaching, increased rate of water loss	Enhanced sensitivity to drought	[148]
*Cucumis sativus*	*CsCER1*	Increased very-long-chain alkanes biosynthesis	Increased drought resistance	[149]
*Arabidopsis thaliana*	*CER1*	Increased very-long-chain alkanes biosynthesis	Increased abiotic stresses resistance	[150]
*Brassica napus*	*BnLAS*	Increased epidermal wax deposition	Improved drought tolerance	[151]
*Arabidopsis thaliana*	*DEWAX*	Reduction in total wax loads in leaves and stems and altered the ultrastructure of cuticular layers.	Decreased drought tolerance	[152]
*Arabidopsis thaliana*	*CER9*	Increased C18 cutin monomers, and very-long-chain free fatty acids, tetracosanoic acid (C24) and hexacosanoic acid (C26).	Increase water status and drought stress	[153]
*Oryza sativa*	*DROUGHT* *HYPERSENSITIVE (DHS)*	Reduced wax loads by overexpression of gene and increased in the mutant genotypes	Decreased drought tolerance in the overexpressed plant and increased in the mutant plant	[154]
*Triticum aestivum*	*TaSHN1*	Increased accumulation of alkanes and reduced stomatal density	Increased drought stress tolerance	[155]
*Arabidopsis thaliana*	*DEWAX2 (dewax2mutant)*	Increased total wax loads in the mutant plant	Increased drought tolerance in the mutant plant	[156]
*Brachypodium distachyon*	*BdFAR*	Increased cuticular wax accumulation, especially primary alcohol	Increased drought and cold stress	[157]
*Oryza sativa*	*Leaf Gas Film 1 (LGF1)*	Increased C30 primary alcohol synthesis and wax platelet	Increased leaf hydrophobicity, gas film retention, submergence and waterlogging tolerance	[158]
*Arabidopsis thaliana*	*WINL1*	Increased the accumulation of wax and cutin	Enhanced drought tolerance	[159]
*Triticum aestivum*	*WXPL*	Increased cuticle biosynthesis	Increased drought tolerance	[160]

## Data Availability

Not applicable.
